# Advanced biomaterials and digital twins for precision psychiatry: neuroimmune modulation and AI-guided therapeutics

**DOI:** 10.3389/fbioe.2026.1917260

**Published:** 2026-08-26

**Authors:** Wentao Wu, Xin Xu, Jiaxin Hu, Jiali He, Mingzhe Yang, Fei Wen

**Affiliations:** The Second Affiliated Hospital of Guangzhou Medical University, Guangzhou, China

**Keywords:** advanced biomaterials, artificial intelligence, bioelectronic medicine, depression, digital twins, neuroimmune modulation, precision psychiatry, psychiatric disorders

## Abstract

Precision psychiatry should be reframed as an integrated bioengineering problem—not simply as a computational challenge of improving diagnostic classification or treatment prediction. In this Perspective, we argue that the next stage of precision psychiatry must move beyond symptom-based stratification and algorithmic prediction toward biologically informed therapeutic engineering. We propose that advanced biomaterials should be viewed not merely as drug carriers or device components, but as programmable therapeutic interfaces capable of modulating neuroimmune and neurochemical microenvironments. Similarly, digital twins should not be treated only as predictive dashboards, but as patient-specific models that connect multimodal data with treatment selection, biomaterial design, neuromodulation parameters, and longitudinal outcome evaluation. Psychiatric disorders—including major depressive disorder, schizophrenia, bipolar disorder, anxiety disorders, obsessive-compulsive disorder, and substance use disorder—increasingly appear to involve dynamic interactions among neural circuits, immune signaling, endocrine stress responses, blood-brain barrier function, synaptic remodeling, gut-brain communication, sleep-wake rhythms, and environmental exposures. These multidimensional processes explain why patients with similar diagnoses follow different trajectories and respond differently to the same interventions. Future progress depends on three converging priorities: moving from diagnostic categorization to dynamic neuroimmune state modeling; redefining biomaterials as active modulators of psychiatric disease biology rather than passive delivery platforms; and evaluating digital psychiatry not only by predictive accuracy, but by its capacity to guide safe, mechanistically grounded, and clinically executable interventions. This framework may help reposition precision psychiatry as a translational field in which computational modeling and material-based therapeutics are developed together to support individualized mental health intervention.

## 1 Introduction

Psychiatric disorders remain among the most disabling and difficult-to-treat conditions in medicine. Major depressive disorder, schizophrenia, bipolar disorder, anxiety disorders, obsessive-compulsive disorder, and substance use disorder collectively affect cognition, emotion, behavior, interpersonal function, physical health, and long-term social participation. Despite substantial progress in pharmacotherapy, psychotherapy, neuromodulation, and community-based care, clinical management remains dominated by symptom-based classification and trial-and-error treatment selection (Rosendal et al., 2017). A patient is usually diagnosed according to clinical interviews and standardized criteria, treated with a relatively limited set of pharmacological or behavioral interventions, and reassessed episodically over weeks or months. This approach is practical and clinically necessary, but it often fails to capture the biological heterogeneity and temporal instability that characterize many psychiatric disorders (Epstein et al., 2016).

The limitations of this paradigm are increasingly visible. Patients with the same diagnosis may differ markedly in inflammatory profiles, stress physiology, sleep rhythm, brain connectivity, cognitive impairment, medication sensitivity, relapse risk, and functional outcome. Conversely, patients with different diagnostic labels may share overlapping biological features, such as chronic low-grade inflammation, altered microglial activity, hypothalamic–pituitary–adrenal axis dysregulation, synaptic plasticity disruption, metabolic dysfunction, or gut–brain axis perturbation (Mencacci et al., 2020). These observations do not mean that current diagnostic systems are useless. Rather, they suggest that diagnostic categories alone are too coarse to guide the next-generation of personalized interventions.

Precision psychiatry has emerged in response to this problem. Much of the field has focused on using artificial intelligence and machine learning to integrate clinical, neuroimaging, genetic, electrophysiological, digital phenotyping, and wearable data in order to improve risk prediction, disease stratification, and treatment-response modeling (Lin et al., 2020). These efforts are valuable because psychiatric disorders are multidimensional, and no single biomarker has yet proven sufficient for routine clinical decision-making. However, a computationally centered version of precision psychiatry also has limitations. A model that predicts relapse, treatment resistance, or symptom severity does not automatically create a better intervention. Similarly, an algorithm that identifies a patient subtype may remain clinically inert unless it can be linked to an actionable therapeutic strategy (Del Casale et al., 2023).

This is where bioengineering becomes important. Psychiatric disorders have rarely been discussed as targets for advanced biomaterials, except in the context of drug delivery, neural implants, or neurodegenerative comorbidity (Chaki, 2025). Yet many disease-relevant processes in psychiatry are material and interface problems in a broad biological sense. The blood–brain barrier regulates access between peripheral immune signals and the central nervous system. The extracellular matrix influences synaptic plasticity and perineuronal net remodeling. Microglia and astrocytes shape inflammatory and trophic microenvironments. Injectable, implantable, wearable, and nanoscale materials can interact with these systems by delivering therapeutic molecules, sensing biological changes, modulating immune responses, or supporting bioelectronic communication (Gómez-Carrillo et al., 2023). Therefore, advanced biomaterials may provide the missing therapeutic arm of precision psychiatry: a way to translate computationally identified disease states into targeted biological modulation.

**FIGURE 1:**
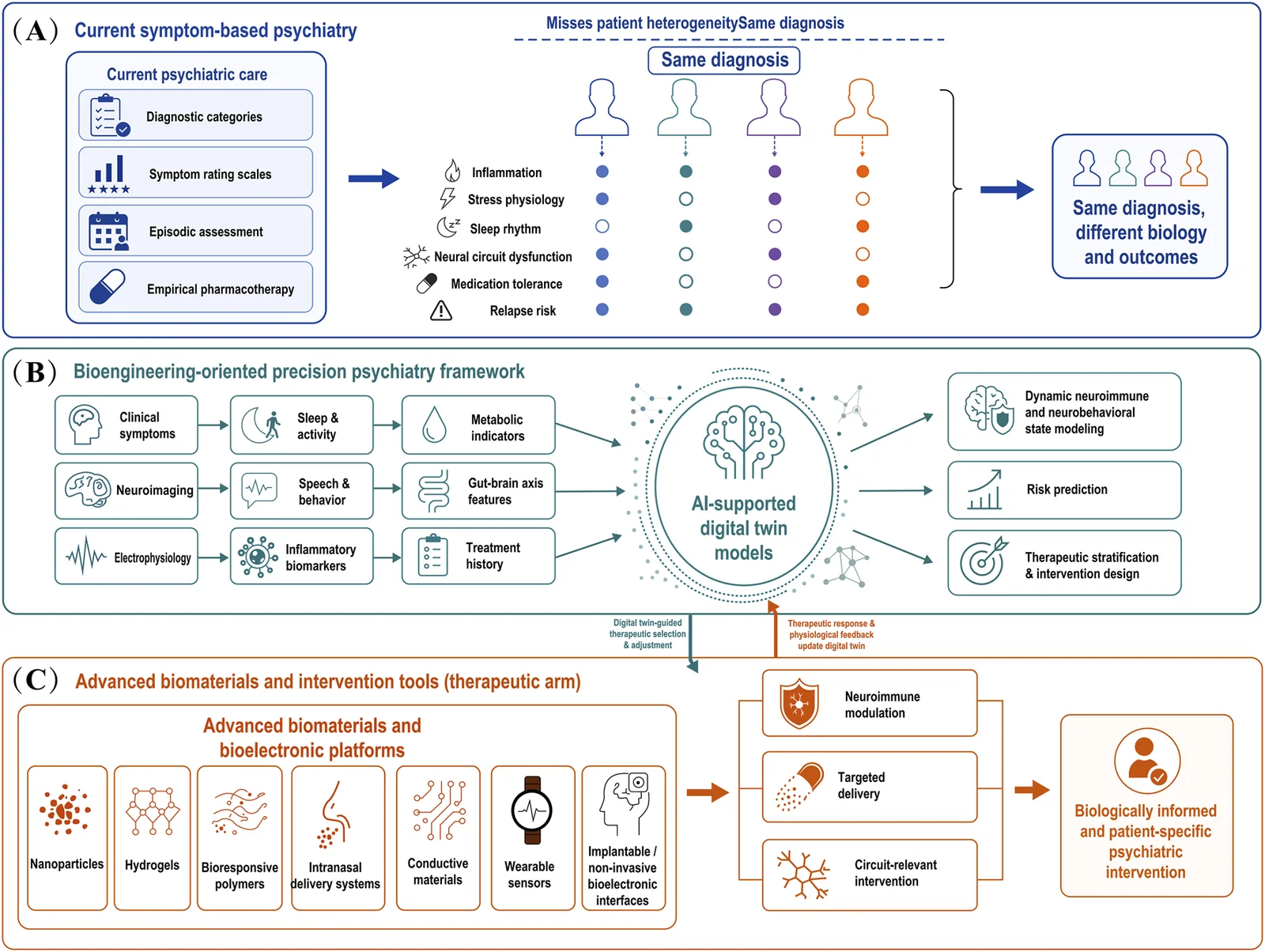
From symptom-based psychiatry to a closed-loop bioengineered precision psychiatry framework. **(A)** Current psychiatric care is primarily organized around diagnostic categories, symptom rating scales, episodic assessment, and empirical pharmacotherapy. This approach may overlook substantial biological and clinical heterogeneity among patients with the same diagnosis, including differences in inflammation, stress physiology, sleep rhythm, neural circuit dysfunction, medication tolerance, and relapse risk. **(B)** In the proposed framework, multimodal clinical, behavioral, physiological, imaging, and biological data are integrated into AI-supported digital twin models to characterize dynamic neuroimmune and neurobehavioral states, predict risk, and guide therapeutic stratification and intervention design. **(C)** Advanced biomaterials and bioelectronic platforms—including nanoparticles, hydrogels, bioresponsive polymers, intranasal delivery systems, conductive materials, wearable sensors, and implantable or non-invasive interfaces—support neuroimmune modulation, targeted delivery, and circuit-relevant intervention. Importantly, **(B,C)** form a bidirectional closed loop: digital twin-derived state estimates guide therapeutic selection and adjustment, whereas treatment responses, physiological signals, safety information, and clinical outcomes feed back into the digital twin for continuous updating and recalibration, thereby enabling biologically informed and patient-specific psychiatric intervention.

In this Perspective, we propose that precision psychiatry should be reframed as an integrated bioengineering problem. The goal is not simply to make psychiatric diagnosis more data-rich or to develop new biomaterials in isolation. Rather, the goal is to connect patient-specific disease modeling with programmable therapeutic interfaces that can modulate neuroimmune, neurochemical, and circuit-level dysfunction. We focus on advanced biomaterials and digital twins because they represent two complementary directions. Biomaterials can intervene in biological systems, while digital twins can model patient-specific trajectories and therapeutic possibilities. When developed together, they may support a more mechanistically grounded and clinically executable form of precision mental health intervention (Figure 1).

To advance this vision, we propose that future progress in precision psychiatry depends on three converging priorities. The first is neuroimmune state modeling: psychiatric disorders should be studied not only as diagnostic entities, but as evolving biological and behavioral states shaped by immune, neural, endocrine, metabolic, and environmental interactions. The second is therapeutic interface design: advanced biomaterials should be developed as active modulators of psychiatric disease biology, with attention to delivery, responsiveness, biocompatibility, and mechanism. The third is translational discipline: digital twins and AI-guided therapeutics should be judged by clinical actionability, prospective validation, safety, equity, and patient acceptability, rather than by retrospective predictive performance alone. These three priorities serve as the organizational backbone of this Perspective. In Sections 2–4, we elaborate on the conceptual foundations of each priority—dynamic neuroimmune state modeling, biomaterials as therapeutic interfaces, and digital twins as treatment-guiding tools. In Section 5, we examine how these priorities apply across representative psychiatric disorders. In Sections 6–7, we discuss the translational requirements and future directions for co-developing materials, models, and clinical endpoints. Finally, in Section 8, we synthesize these themes and reflect on what the framework implies for the field as a whole.

Because this article is a Perspective rather than an experimental or computational methods study, it does not report newly synthesized biomaterials, original biological experiments, clinical datasets, or executable artificial intelligence code. Its contribution is to define a testable translational framework for connecting patient-specific state modeling with biomaterial-assisted and bioelectronic interventions. Accordingly, the proposed integration should be interpreted as a research agenda requiring prospective biological, computational, safety, and clinical validation, rather than as an immediately deployable therapeutic platform.

## 2 Precision psychiatry should model dynamic neuroimmune states, not only diagnostic categories

Psychiatric disorders have historically been organized around symptoms, behavior, and functional impairment. This clinical framework remains essential because psychiatric diagnosis must be interpretable, communicable, and applicable in real-world care. However, symptom categories do not map neatly onto biological mechanisms. Major depressive disorder may include patients with prominent inflammation, stress-axis dysregulation, metabolic dysfunction, trauma-related hyperarousal, circadian disruption, or neuroplasticity impairment (Bai et al., 2025). Schizophrenia may involve neurodevelopmental vulnerability, aberrant synaptic pruning, dopaminergic dysregulation, cognitive network dysfunction, immune activation, and metabolic comorbidity (Keshavan et al., 2020). Bipolar disorder may be characterized by state transitions between depression, euthymia, hypomania, mania, and mixed states, each potentially associated with different circadian, inflammatory, and neurochemical profiles (Spielberg et al., 2016). Substance use disorder may involve reward-circuit adaptation, stress sensitization, neuroimmune activation, and relapse triggered by environmental cues (van Holst et al., 2017). These examples illustrate why static diagnostic labels are not sufficient as the sole organizing principle for precision intervention.

A more useful framework may be to view psychiatric disorders as dynamic neuroimmune and neurobehavioral systems. In this view, symptoms emerge from interactions among neural circuits, immune mediators, endocrine responses, metabolic state, sleep–wake rhythms, social context, and environmental stress (Kaštelan et al., 2025). Neuroimmune signaling is particularly important because it provides a mechanistic bridge between the brain and systemic physiology. Cytokines can influence neurotransmitter metabolism, synaptic plasticity, neuroendocrine function, sickness behavior, motivation, and cognition (Samadzadeh et al., 2025). Microglia can contribute to synaptic pruning, inflammatory amplification, and neuroplastic remodeling. Astrocytes regulate metabolic support, glutamate homeostasis, blood–brain barrier function, and inflammatory signaling. Peripheral immune activation can influence central circuits through humoral, neural, and barrier-mediated routes (Kobrzycka et al., 2019). Although evidence from neurological autoimmune disorders cannot be directly extrapolated to psychiatric disease, research in multiple sclerosis demonstrates that B cells can participate in central nervous system inflammation through antigen presentation, cytokine secretion, and meningeal immune activity, providing a relevant example of peripheral–central immune crosstalk (Jiang et al., 2024a). These processes may vary over time, producing fluctuating disease states rather than fixed biological traits.

This shift has direct implications for therapeutic design. If psychiatric disorders are treated only as stable diagnostic categories, interventions are likely to remain standardized and symptom-driven. If they are treated as dynamic neuroimmune states, therapy can be designed around measurable biological and behavioral trajectories. For example, an inflammatory subtype of depression may require different adjunctive strategies than a patient whose dominant features involve circadian disruption and stress sensitivity (Miller, 2025). A patient with bipolar disorder approaching a manic transition may require intervention based on sleep, activity, and physiological warning signals rather than retrospective symptom reporting alone (Kragh et al., 2026). A patient with substance use disorder may require relapse prevention strategies that account for stress biomarkers, environmental exposure, craving dynamics, and reward-circuit vulnerability (Abroms et al., 2019). In each case, precision depends not only on identifying the diagnosis, but on modeling the state of the system.

Digital twins are conceptually well suited to this task. A psychiatric digital twin can be understood as a computational representation of an individual patient’s evolving mental, behavioral, and biological state (Spitzer et al., 2023). Unlike a one-time risk model, a digital twin is designed to integrate data across time and update as new information becomes available. In psychiatry, such data may include clinical symptoms, medication history, adverse effects, sleep patterns, physical activity, speech features, cognitive testing, neuroimaging, electroencephalography, inflammatory markers, metabolic indicators, hormonal rhythms, gut microbiome profiles, and contextual information. The value of this approach is not that every patient must be measured with every modality, but that the model can be organized around patient-specific trajectories rather than population averages alone (Yu et al., 2026).

However, digital twins should not become another layer of passive monitoring. Their translational value depends on whether they can guide intervention. A model that identifies a neuroimmune state should help determine whether a patient might benefit from anti-inflammatory modulation, neurotrophic support, circadian stabilization, neuromodulation, medication adjustment, behavioral intervention, or biomaterial-assisted delivery. The central question is therefore not only whether psychiatric digital twins can predict outcomes, but whether they can connect biological state modeling with therapeutic engineering.

## 3 Biomaterials should be redefined as active therapeutic interfaces for psychiatric biology

Advanced biomaterials have transformed many areas of regenerative medicine, oncology, immunotherapy, wound healing, and central nervous system repair. In psychiatry, their role has been more limited and is often reduced to drug delivery (Hamidi et al., 2026). This narrow view underestimates their potential. Biomaterials can be designed to control spatial distribution, release kinetics, immune interaction, electrical conductivity, mechanical compatibility, molecular presentation, and biological responsiveness (Zhang et al., 2022). These properties are directly relevant to psychiatric disorders when the target is not merely neurotransmitter concentration, but the broader neuroimmune and neurochemical microenvironment.

The first major role of biomaterials in precision psychiatry is targeted and sustained delivery (Tesauro et al., 2019). Many psychiatric drugs are limited by systemic exposure, delayed onset, off-target effects, poor adherence, and difficulty maintaining optimal therapeutic windows. Long-acting injectable formulations already demonstrate the value of delivery engineering in psychiatric care, especially for antipsychotic treatment (Uvais, 2018). However, future delivery systems could go further by targeting specific biological pathways, improving central nervous system access, or enabling controlled release of molecules that are not easily delivered by conventional routes. Nanoparticles, liposomes, polymeric carriers, exosome-mimetic vesicles, and intranasal delivery platforms may be used to transport anti-inflammatory agents, neurotrophic modulators, nucleic acids, peptides, or small molecules across biological barriers. For psychiatric disorders with neuroimmune components, such systems could theoretically reduce systemic toxicity while improving local biological relevance.

Neuroimmune modulation should not, however, be interpreted as the sole biological basis of biomaterial-assisted psychiatry. Glutamatergic and GABAergic dysfunction, dopaminergic and serotonergic signaling abnormalities, impaired synaptic plasticity, and circuit-level dysregulation are also central to many psychiatric disorders. These mechanisms may interact with inflammatory and glial pathways but cannot be reduced to them. Compared with anti-inflammatory delivery, biomaterial strategies directly targeting excitatory–inhibitory neurotransmission remain substantially less developed. In particular, nano-enabled delivery of antibodies or enzymes to correct glutamatergic or GABAergic dysfunction has not been established in psychiatric models. Such approaches should therefore be regarded as an important research gap requiring validation of target engagement, blood–brain barrier transport, circuit specificity, reversibility, and neuropsychiatric safety, rather than as currently available therapeutic options.

The second role is microenvironment-responsive modulation. Psychiatric disease biology includes oxidative stress, inflammatory signaling, metabolic disturbance, and stress-related endocrine changes. Responsive biomaterials can be engineered to release therapeutic agents in response to reactive oxygen species, pH changes, enzymatic activity, inflammatory mediators, or other pathological cues (Ren et al., 2022). In depression or substance use disorder, where inflammatory and oxidative pathways may contribute to treatment resistance or relapse vulnerability, such materials could support state-dependent therapy. In schizophrenia or bipolar disorder, responsive systems might be developed to modulate neuroinflammatory or metabolic components that fluctuate across disease stages. Although this remains largely conceptual for psychiatry, related principles are already being explored in broader central nervous system disease, injury, and inflammation contexts.

The third role is bioelectronic interfacing. Many psychiatric interventions ultimately aim to influence neural circuits. Existing neuromodulation approaches, including transcranial magnetic stimulation, electroconvulsive therapy, vagus nerve stimulation, deep brain stimulation, and transcranial direct current stimulation, demonstrate that circuit modulation can produce meaningful psychiatric effects (Mahoney et al., 2020). Yet current approaches often face limitations in personalization, tolerability, anatomical targeting, and long-term adaptation. Biomaterials can contribute through flexible electrodes, conductive hydrogels, stretchable electronics, neural probe coatings, wearable sensors, and implantable interfaces that reduce tissue mismatch and improve biocompatibility (Hernandez and Woodrow, 2022). These materials may allow more stable signal acquisition, safer stimulation, and better integration between physiological sensing and therapeutic output.

The fourth role is immune and glial modulation. In the central nervous system, the interface between implanted or injected materials and tissue is strongly shaped by microglia, astrocytes, macrophages, extracellular matrix remodeling, and vascular responses (Hsieh et al., 2015). Rather than treating these responses only as obstacles, next-generation materials could be designed to influence them. Anti-inflammatory coatings, immunomodulatory hydrogels, extracellular vesicle-based carriers, and matrix-mimetic materials may help regulate local neuroimmune tone (Tang et al., 2020). This is particularly relevant when maladaptive neuroimmune regulation coexists with neurotransmitter, synaptic, and circuit-level dysfunction; these mechanisms should be viewed as interacting rather than mutually exclusive.

For these reasons, biomaterials in psychiatry should not be viewed simply as vehicles for old drugs. They should be regarded as therapeutic interfaces that can translate patient-specific biological information into controlled intervention. This reframing changes the criteria for material design. A psychiatric biomaterial should not be judged only by loading efficiency, release profile, or mechanical performance. It should also be evaluated by whether it modulates disease-relevant neuroimmune pathways, preserves safety in vulnerable neural systems, integrates with patient-specific treatment models, and produces clinically meaningful changes in symptoms, function, relapse risk, or treatment tolerability.

## 4 Digital twins should guide therapeutic design, not only risk prediction

The rise of artificial intelligence in psychiatry has generated considerable enthusiasm. Machine learning models have been applied to neuroimaging, electronic health records, genomics, speech, facial expression, smartphone behavior, wearable data, and social interaction patterns (Su et al., 2020; Bernstorff et al., 2022; Nainwal et al., 2025; Bhamare and Ashok Kumar, 2026). These models may help identify risk states, predict relapse, estimate treatment response, and stratify heterogeneous patient populations. However, the field has also faced concerns about small sample sizes, poor external validation, algorithmic bias, limited interpretability, data privacy, and weak clinical implementation. A high-performing model in a retrospective dataset may not improve care if it cannot be trusted, explained, validated, or connected to a real therapeutic decision (Crockett et al., 2023).

Before proceeding, it is useful to clarify what we mean by a psychiatric digital twin and how it differs from conventional predictive models. A digital twin, as we use the term here, is a computational representation of an individual patient’s evolving state that is not merely predictive but interventional in purpose. Unlike a longitudinal Bayesian state-space model or a recurrent neural network trained solely to forecast relapse risk or symptom severity, a digital twin is designed to simulate counterfactual scenarios—e.g., how would this patient’s depressive trajectory change if we initiated an anti-inflammatory biomaterial intervention?—and to update its predictions as new clinical and biological data arrive (Spitzer et al., 2023). Operationally, we define a psychiatric digital twin by three features: (a) it is calibrated to an individual’s baseline characteristics rather than population averages; (b) it is bidirectionally coupled to the patient, continuously assimilating new data streams; and (c) its outputs are framed as actionable therapeutic decisions rather than abstract risk probabilities. We acknowledge that fully realized psychiatric digital twins do not yet exist; early implementations are likely to be modular and task-specific, but what makes them “digital twins” rather than conventional classifiers is their explicit design for intervention testing and continuous clinical feedback.

Because this Perspective does not present an implemented computational system, executable model code is not provided. Nevertheless, any future psychiatric digital twin should meet several minimum operational requirements: patient-specific baseline calibration, longitudinal assimilation of multimodal data, explicit uncertainty quantification, counterfactual simulation of alternative interventions, clinically interpretable and action-oriented outputs, and prospective recalibration against observed treatment responses. Models that do not satisfy these requirements should be regarded as longitudinal prediction tools rather than fully operational digital twins.

In practice, missing data should be handled according to their likely mechanism, duration, and clinical relevance. Short and plausibly random gaps may be addressed using transparent statistical imputation, whereas prolonged or potentially informative missingness—such as several consecutive days of wearable non-use or device failure—should prompt greater reliance on available alternative data streams and explicit propagation of increased uncertainty. The model should distinguish imputed observations from directly measured data and record any substitutions or uncertainty adjustments. When data coverage or model confidence falls below predefined thresholds, treatment recommendations should be temporarily withheld and the case escalated for clinician review rather than inferred from low-confidence estimates. The patient state should then be recalibrated when reliable new observations become available.

Digital twins offer a more clinically oriented way to think about AI in psychiatry. Instead of treating machine learning as a standalone classifier, a digital twin can incorporate multiple forms of patient data into an evolving model of disease state and intervention response (Wen, 2025). This is especially important because psychiatric disorders unfold over time. A patient’s risk of depressive relapse, manic switch, psychotic exacerbation, or substance-use relapse is rarely determined by one variable. It may arise from interactions among medication adherence, sleep disruption, inflammatory state, stress exposure, social rhythm, cognitive load, substance cues, and prior disease history. A useful digital twin should therefore represent trajectories, transitions, and modifiable mechanisms rather than static categories alone.

For biomaterial-based psychiatry, digital twins could play several roles (Zhang et al., 2025). First, they could identify which patients are biologically appropriate for a given intervention. A patient with depression and elevated inflammatory markers may be a more plausible candidate for immunomodulatory adjunctive therapy than a patient without evidence of inflammatory involvement. A patient with schizophrenia and prominent cognitive decline may require a different strategy than one with predominantly positive symptoms and good functional recovery (Chen et al., 2026). Second, digital twins could guide dosing, timing, and delivery routes. Biomaterial-assisted delivery systems may require decisions about release duration, target tissue, frequency of administration, and combination with conventional drugs or neuromodulation (Abdullah et al., 2026). Third, digital twins could support safety monitoring by integrating symptom change, adverse events, physiological signals, and biomarkers. Fourth, they could help generate mechanistic hypotheses by linking patient-level data to biological responses.

This approach also changes the meaning of validation. In conventional AI psychiatry, validation often focuses on predictive accuracy (Sun et al., 2025). Experience from other clinical fields has shown that deep learning models can integrate multiple prognostic variables, outperform conventional nomograms, and be translated into web-based tools for individualized outcome estimation, although such systems remain primarily predictive rather than interventional (Zhang et al., 2023). In a bioengineering framework, validation must also ask whether the model improves intervention selection, reduces unnecessary exposure, enhances therapeutic durability, identifies adverse trajectories earlier, or supports better functional outcomes. A digital twin that predicts relapse but does not change management may have limited translational value. Conversely, a model with modest predictive performance may be clinically valuable if it helps guide safe and actionable treatment adjustments.

A realistic psychiatric digital twin does not need to be a perfect simulation of the entire brain. Such an expectation would be scientifically unrealistic and clinically unnecessary. Instead, early models may be modular and task-specific. One digital twin may focus on depressive relapse risk using sleep, activity, mood, and inflammatory data (Mahadevan et al., 2021). Another may model bipolar state transitions using circadian rhythm, medication history, and behavioral variability (Alloy et al., 2015). Another may support substance-use relapse prevention using craving reports, stress physiology, geolocation risk patterns, and prior relapse triggers (Sinha, 2024). Another may guide neuromodulation parameters using electrophysiology and symptom response. The important point is that the model should be clinically interpretable, updateable, and linked to intervention.

## 5 Representative psychiatric contexts require different engineering logics

The three priorities outlined above—neuroimmune state modeling, therapeutic interface design, and translational discipline—apply across psychiatric disorders, but their implementation must be tailored to the distinct biology, time course, and clinical challenges of each condition. In this section, we briefly illustrate how these engineering logics differ across representative disorders, focusing on disease-specific features that shape biomaterial and digital twin design. A structured overview is provided in Table 1.

**TABLE 1:** Representative psychiatric contexts and bioengineering opportunities for AI-guided precision intervention.

Psychiatric context	Dynamic biological and clinical features	Potential biomaterial strategies	Potential digital twin or AI-guided role	Key translational concerns
Major depressive disorder	Inflammatory subtype, HPA-axis dysregulation, impaired neuroplasticity, sleep disturbance, fatigue, treatment resistance	Anti-inflammatory delivery systems, neurotrophic factor modulation, intranasal delivery, responsive hydrogels, adjunctive bioelectronic interfaces	Identify inflammatory or circadian subtypes, estimate treatment response, guide adjunctive therapy, monitor relapse risk	Avoid overgeneralizing inflammation; validate biomarkers; ensure safety of CNS-targeted delivery
Schizophrenia	Neurodevelopmental vulnerability, synaptic dysfunction, cognitive impairment, relapse risk, metabolic comorbidity, possible immune activation	Long-acting drug delivery, targeted nanoparticles, neuroimmune modulation, neural interface materials, adherence-supporting formulations	Model disease stage, relapse risk, cognitive trajectory, medication response, adverse-effect susceptibility	Long-term safety, adherence, metabolic monitoring, patient autonomy, cognitive endpoints
Bipolar disorder	Mood-state transitions, circadian disruption, sleep instability, inflammation/metabolic fluctuation, medication sensitivity	Long-acting mood-stabilizing delivery, rhythm-informed release systems, wearable-linked neuromodulation, adjunctive anti-inflammatory systems	Model transition risk between depression, euthymia, hypomania, mania, and mixed states; guide early intervention	Risk of false alarms, state-specific treatment decisions, sleep data quality, ethical use of monitoring
Substance use disorder	Reward-circuit adaptation, craving, withdrawal, stress-triggered relapse, cue exposure, neuroimmune activation	Long-acting medication delivery, anti-inflammatory adjuncts, bioelectronic modulation, wearable-integrated support systems	Predict relapse windows, identify stress/cue-related risk, personalize intervention timing, monitor treatment adherence	Privacy, stigma, coercive monitoring, environmental confounding, patient trust
Anxiety disorders and obsessive-compulsive disorder	Threat processing, autonomic arousal, avoidance, compulsivity, habit formation, cortico-striatal circuit dysfunction	Wearable physiological sensing, bioelectronic interfaces, adjunctive delivery systems, neuromodulation-supporting materials	Model triggers, arousal patterns, avoidance cycles, treatment response to exposure or neuromodulation	Integration with psychotherapy, patient burden, false positives, symptom-context interpretation

Major depressive disorder is a compelling initial context because inflammation, hypothalamic–pituitary–adrenal (HPA) axis dysregulation, and impaired neuroplasticity define a subset of patients who may benefit from immunomodulatory or neurotrophic adjunctive strategies. The key engineering challenge is not simply delivering an antidepressant, but delivering anti-inflammatory or plasticity-promoting agents to the central nervous system (CNS) with minimal systemic exposure—an application for which intranasal formulations, nanoparticle carriers, and hydrogel-based platforms are particularly relevant. Digital twins could help identify inflammatory, circadian, or stress-related subtypes and simulate whether an individual patient is more likely to respond to an anti-inflammatory adjunct than to a conventional antidepressant adjustment. The central opportunity is not to replace existing treatments, but to identify patients for whom material-assisted biological modulation could complement pharmacotherapy. This vision is increasingly grounded in tangible evidence. Clinically, the FDA-approved intranasal esketamine (SPRAVATO®) for treatment-resistant depression has demonstrated that non-invasive CNS delivery via the nasal route is not merely conceptual but therapeutically viable in psychiatric practice. However, the clinical feasibility of an intranasally administered small molecule should not be interpreted as direct validation of nanoparticle-mediated nose-to-brain delivery, because complex nanocarriers face distinct challenges related to mucus retention, mucosal penetration, epithelial transport, biodistribution, bioavailability, formulation reproducibility, and long-term safety. Against this background, recent advances have pushed beyond conventional delivery into active neuromodulation: biomineralized silk fibroin nanoparticles (CaSF@TB) for nose-to-brain delivery achieve ∼6.2-fold elevation of hippocampal brain-derived neurotrophic factor (BDNF) expression and significant behavioral improvement (p < 0.0001) in lipopolysaccharide (LPS)-induced depressive mice through synergistic microglial M2 polarization and neuroinflammation elimination, while photochemical nanomotors (IC@His-ICG) enable wireless, near-infrared-responsive spatiotemporal polarity tuning to restore monoamine homeostasis and reverse depressive behaviors in rodents. These examples illustrate that biomaterial strategies for MDD are transitioning from conceptual frameworks to experimentally validated platforms.

Schizophrenia presents a different challenge. Its neurodevelopmental origins, cognitive impairment trajectory, and long-term functional decline demand strategies that prioritize adherence, metabolic safety, and stage-specific intervention. Long-acting injectable formulations have already demonstrated the clinical value of pharmacokinetic engineering in this population. Future materials may extend this logic toward targeted neuromodulation or neuroprotective strategies that address cognitive or negative symptoms—domains poorly served by current antipsychotics. Digital twins could model disease stage, relapse risk, and medication tolerability to help distinguish patients who need relapse prevention from those who need cognitive remediation, enabling more stratified long-term management.

Bipolar disorder is uniquely suited to dynamic modeling because its clinical course is defined by state transitions—depression, euthymia, hypomania, mania, and mixed states—rather than a stable baseline. A static predictive model is insufficient; what is needed is a system that tracks transition risk in near real-time using sleep, activity, circadian, and physiological signals, and that can trigger early intervention before a full affective episode unfolds. Biomaterial strategies in this context might include long-acting mood-stabilizing delivery systems that could be adjusted or complemented based on estimated transition risk. The engineering challenge is temporal: biomaterials with fixed release kinetics must be paired with digital models that recognize when the patient’s state has changed enough to warrant a different therapeutic approach.

Substance use disorder involves reward-circuit adaptation, craving dynamics, stress sensitization, and cue-triggered relapse. Here, the biological vulnerability is not a static trait but a state that fluctuates with environmental exposure, stress, and withdrawal. The engineering priority is to identify relapse windows—periods of heightened risk—and to time interventions accordingly. Long-acting medication delivery (e.g., for opioid or alcohol use disorder) could be paired with wearable-integrated digital twins that monitor physiological arousal, sleep disruption, and craving reports to predict when a patient is entering a high-risk period. The key translational concern is preserving patient autonomy and avoiding coercive monitoring, as substance use populations are particularly sensitive to surveillance-like care models.

Anxiety disorders and obsessive-compulsive disorder are characterized by threat processing dysfunction, autonomic hyperarousal, and cortico-striatal circuit dysregulation. These conditions are often tightly coupled to environmental triggers and psychological processes such as avoidance and habituation. Bioengineering approaches here may emphasize wearable physiological sensing and bioelectronic interfaces that can detect arousal spikes and deliver real-time feedback or neuromodulation. Digital twins could model trigger-response patterns and help personalize exposure-based psychotherapy by predicting which stimuli or contexts are most likely to provoke symptoms. The key challenge is integration with psychotherapy—technology should support, not replace, the therapeutic relationship and behavioral activation that remain central to effective care.

Across these diverse conditions, several cross-cutting themes emerge. First, the temporal mismatch between biomaterial release kinetics (hours to months) and psychiatric state dynamics (days to weeks) must be addressed through responsive materials or combination with digital monitoring. Second, the biological heterogeneity within each diagnostic category means that no single material or model will fit all patients—stratification is essential. Third, the safety bar for psychiatric biomaterials is exceptionally high, as even subtle effects on cognition, emotion, or behavior carry profound implications for patient identity and quality of life. These shared challenges, rather than the disorder-by-disorder details, will ultimately determine whether the proposed framework translates into clinical practice.2.

## 6 Translation should be judged by clinical executability, not technological sophistication alone

The convergence of biomaterials, AI, and digital twins is attractive, but it also carries a risk of overstatement. Psychiatry has seen repeated waves of technological optimism, from neuroimaging biomarkers to mobile mental health applications and algorithmic prediction tools (Richter et al., 2025). Many have generated important knowledge, but relatively few have transformed routine clinical care. A bioengineering approach to precision psychiatry must therefore be judged not by conceptual elegance alone, but by clinical executability.

The first translational requirement is biological validation. If a biomaterial is designed to modulate neuroimmune signaling, it should be evaluated in models that measure disease-relevant immune, glial, synaptic, behavioral, and safety endpoints. For psychiatric applications, simple release kinetics or cell viability assays are insufficient (Jiang et al., 2024b). Preclinical studies should assess whether the material influences microglial phenotype, cytokine networks, blood–brain barrier function, synaptic plasticity, neural circuit activity, and behavior in a coherent manner. The goal is not to force every psychiatric disorder into an inflammatory model, but to ensure that the proposed mechanism is biologically plausible and experimentally supported.

The second requirement is material safety. Neural and psychiatric applications demand particular caution because implantable neuromodulation may be associated with cognitive, affective, and behavioral adverse effects, whereas CNS-targeted nanomaterials may introduce neurotoxicity and long-term biocompatibility concerns (Appleby et al., 2007; Yang et al., 2010). Nanoparticles, hydrogels, bioelectronic interfaces, and implantable systems must be assessed for neurotoxicity, immunogenicity, degradation products, off-target distribution, chronic inflammation, and reversibility. Materials that are acceptable in peripheral tissue engineering may not automatically be acceptable for central nervous system or psychiatric applications. In addition, psychiatric patients may require long-term or repeated interventions, making durability and cumulative risk especially important.

Unlike peripheral tissues, the brain parenchyma lacks conventional lymphatic drainage, and the clearance of CNS-delivered materials may involve cellular uptake, perivascular transport, cerebrospinal fluid exchange, and meningeal lymphatic outflow (Louveau et al., 2018; Ahn et al., 2019). The biodistribution and persistence of these materials are therefore strongly influenced by their size, surface chemistry, charge, biodegradability, and route of administration. Poorly degradable nanomaterials may persist within brain tissue or be sequestered by glial and perivascular cells, whereas hydrogels may undergo hydrolytic or enzymatic degradation; in both cases, the degradation kinetics and biological activity of the resulting products require careful characterization. Repeated administration and the use of non-degradable components may increase cumulative tissue burden and prolong inflammatory, oxidative, or off-target effects. Preclinical evaluation should therefore quantify the time-dependent biodistribution, degradation, persistence, and clearance of both the parent material and its degradation products in brain tissue, cerebrospinal fluid, draining cervical lymph nodes, and systemic organs, together with chronic neurobehavioral and immunological safety following repeated exposure.

The third requirement is computational reliability. Digital twins depend on data quality, model assumptions, update frequency, and clinical interpretability (Spitzer et al., 2023). Wearable and smartphone data may be noisy, incomplete, biased by device access, and influenced by non-disease factors (Ali et al., 2023). Biomarker measurements may vary across laboratories and time points. Neuroimaging and electrophysiological data may be expensive or difficult to standardize. Therefore, psychiatric digital twins should be developed with realistic clinical constraints in mind. Models that require dense multimodal data from every patient may be scientifically interesting but difficult to deploy. A tiered strategy may be more practical, in which low-burden data streams such as symptoms, sleep, activity, and medication history are combined with deeper biological profiling when clinically justified.

Measurability in psychiatric digital twins should therefore be treated as a probabilistic and longitudinal problem rather than as a search for a single definitive biomarker. Early models should rely on repeated within-patient measurements and triangulation across complementary data sources, including symptoms, sleep, activity, medication exposure, cognitive performance, physiological signals, and selectively obtained biological markers. Each input should be accompanied by data-quality indicators, while missing or discordant measurements should be reflected in explicit uncertainty estimates rather than being treated as reliable observations. When model confidence falls below a predefined threshold, treatment recommendations should be withheld or escalated for clinician review. The estimated patient state should also be recalibrated against subsequent clinical assessments and observed treatment responses. In this framework, subjective reports remain clinically essential but are treated as one data stream among several, rather than as the sole ground truth.

The fourth requirement is therapeutic actionability. A digital twin should not simply inform the clinician that a patient is high risk. It should help clarify what can be done differently. Does the model suggest a change in medication? A need for closer monitoring? A biomaterial-assisted delivery strategy? A neuromodulation adjustment? A behavioral intervention? A biomarker reassessment? A safety warning? Without actionable pathways, digital twin models risk becoming sophisticated but clinically passive tools.

The fifth requirement is ethical governance. Precision psychiatry involves highly sensitive data, including mood, behavior, sleep, location, speech, cognition, medication adherence, substance use, and possibly biological biomarkers (Munk-Olsen, 2025). Patients must understand how data are collected, stored, interpreted, and used (Bhasha and Panda, 2024). Algorithmic recommendations should not replace clinical judgment or patient preference. Special care is needed to avoid surveillance-like care models, coercive monitoring, insurance misuse, or stigmatizing risk prediction. For biomaterial and bioelectronic interventions, informed consent must address not only procedural risks, but also uncertainty regarding long-term neuropsychiatric effects.

The sixth requirement is health-economic feasibility. Highly individualized digital twin systems combined with customized biomaterial or bioelectronic interventions may generate substantial costs related to multimodal data acquisition, computational infrastructure, material fabrication, regulatory evaluation, specialist training, maintenance, and longitudinal monitoring. Their translational value should therefore be assessed not only by technical performance, but also by cost-effectiveness, scalability, reimbursement feasibility, and the extent to which they improve clinically meaningful outcomes relative to standard care. A tiered implementation strategy may improve accessibility, with lower-cost core models based on routinely collected clinical and behavioral data and more resource-intensive biological, imaging, or material-assisted interventions reserved for patients with clear clinical indications. Shared computational infrastructure, standardized manufacturing processes, interoperable data systems, and health-technology assessment conducted early in development may further reduce implementation costs. Without such measures, these technologies could widen existing disparities in mental healthcare by concentrating access in well-resourced centers. Future development should therefore include evaluation across healthcare settings with different levels of infrastructure and explicitly consider equitable access, affordability, and reimbursement from the earliest stages of design.

Ethical evaluation should also be technology-specific. For implantable bioelectronic interfaces, key considerations include procedural invasiveness, reversibility and explantability, long-term maintenance, device failure, cybersecurity, and possible effects on agency, identity, cognition, or decision-making. For nanoparticles and other non-retrievable materials, major concerns include uncertain biodistribution, persistence, clearance, cumulative exposure, off-target effects, and the limited ability to reverse exposure after administration. In both cases, clinical use should be guided by proportionality and the least-invasive-alternative principle, with enhanced safeguards for patients whose decision-making capacity may fluctuate. Consent should be longitudinal rather than treated as a one-time event, particularly when device settings, algorithms, or material formulations are modified. Governance frameworks should also define responsibility for long-term monitoring, adverse-event management, treatment discontinuation or device removal, post-trial care, and access to intervention-related data. These ethical requirements should be incorporated into material and trial design from the outset rather than treated as downstream regulatory considerations.

## 7 Future progress requires co-development of materials, models, and psychiatric endpoints

The most important future direction is co-development. Biomaterials, digital twins, and psychiatric endpoints should not be developed separately and connected only at the end. If a material is intended for neuroimmune modulation, digital models should help define which neuroimmune states are clinically meaningful. If a digital twin identifies a patient subtype, material scientists should ask what intervention could plausibly modify that subtype. If a clinical endpoint is chosen, it should reflect not only symptom change but also functional recovery, relapse prevention, treatment tolerability, and biological mechanism.

This requires a change in how studies are designed. Early-stage biomaterial studies for psychiatric applications should include clinically relevant biological endpoints, such as inflammatory markers, stress-axis measures, synaptic plasticity indicators, electrophysiological changes, and behavior (Demiroglu et al., 2012). Digital twin studies should include intervention-oriented outputs rather than only classification metrics. Clinical studies should evaluate whether model-guided or material-assisted strategies improve decisions compared with standard care. Regulatory planning should begin early, particularly for combination products that integrate materials, devices, algorithms, and therapeutic agents.

A second priority is disease-stage specificity. Psychiatric disorders evolve over time. The biological and clinical needs of a first-episode psychosis patient differ from those of a patient with chronic schizophrenia and metabolic comorbidity (Chan et al., 2018). A patient with early recurrent depression may differ from one with treatment-resistant depression and chronic inflammation. A patient with bipolar disorder in euthymia may need relapse prevention, while a patient in acute mania needs rapid stabilization. Biomaterials and digital twins should therefore be designed for specific stages and use cases, not for broad diagnostic labels alone.

A third priority is multimodal but practical phenotyping. The ideal precision psychiatry model would integrate molecular, imaging, electrophysiological, behavioral, environmental, and clinical data (Cevoli et al., 2026). In reality, most healthcare systems cannot collect all of these data routinely. Future frameworks should distinguish between core data, optional enrichment data, and research-level data. Core data might include symptoms, medication history, sleep, activity, and adverse effects. Optional enrichment might include inflammatory markers, metabolic markers, EEG, or cognitive testing. Research-level data might include advanced neuroimaging, multi-omics, microbiome profiling, or dense ecological momentary assessment. This tiered approach may make digital twins more clinically scalable.

A fourth priority is patient-centered implementation. Psychiatric care depends heavily on therapeutic alliance, trust, autonomy, and subjective experience. Bioengineering tools should support, not replace, these dimensions. A patient-specific model may help a clinician and patient understand why certain symptoms worsen under stress or sleep disruption. A long-acting material may reduce adherence burden. A wearable sensor may identify early warning signs. A bioelectronic intervention may offer an alternative for treatment-resistant symptoms. But these tools must be integrated into care in ways that patients find acceptable, understandable, and beneficial.

## 8 Discussion

Precision psychiatry is approaching a conceptual crossroads. One path continues to emphasize prediction: better algorithms, larger datasets, more biomarkers, and more refined stratification. This path is necessary, but incomplete. The other path asks how prediction can be translated into intervention. We argue that advanced biomaterials and digital twins provide a way to connect these two directions. Biomaterials offer programmable therapeutic interfaces capable of modulating neuroimmune, neurochemical, and circuit-relevant processes. Digital twins offer patient-specific models capable of integrating multimodal data and simulating disease trajectories or treatment responses. Together, they may help shift psychiatry from symptom-guided trial-and-error care toward biologically informed therapeutic engineering.

The central argument of this Perspective is not that biomaterials or digital twins will replace existing psychiatric care. Pharmacotherapy, psychotherapy, psychosocial support, neuromodulation, and clinician–patient relationships remain indispensable. Rather, the argument is that these existing approaches can be strengthened by bioengineering tools that make psychiatric intervention more individualized, mechanistically grounded, and dynamically informed. The field should therefore avoid both technological reductionism and therapeutic pessimism. Psychiatric disorders are too complex to be solved by a single material, algorithm, biomarker, or device. But they are also too dynamic and heterogeneous to be managed indefinitely by static categories and delayed clinical feedback alone.

Having established the three converging priorities—neuroimmune state modeling, therapeutic interface design, and translational discipline—in the Introduction and developed them across the preceding sections, we now turn to what is learned by considering these themes together. The framework we have outlined is not simply an assembly of three independent agendas. Rather, its value lies in the synergy between them: dynamic state modeling identifies which patients are biologically appropriate for material-based intervention; therapeutic interface design provides the tools to translate that identification into controlled biological modulation; and translational discipline ensures that the resulting strategy is clinically actionable, safe, and acceptable to patients. Conversely, the absence of any one of these priorities would undermine the entire framework—models without materials remain diagnostically inert, materials without models risk being applied to the wrong patients or wrong disease states, and both without translational discipline will fail to reach clinical practice. This interdependence is, in our view, the defining feature of a genuinely bioengineered precision psychiatry.

Several limitations of the proposed framework must be acknowledged. First, the biological mechanisms linking neuroimmune states to psychiatric symptoms remain incompletely understood, and many proposed pathways are correlational rather than causal. Biomaterial strategies designed to modulate these pathways may therefore target the wrong nodes or produce unintended effects. Second, the material safety profile for psychiatric applications is particularly demanding: interventions may affect cognition, emotion, behavior, and long-term brain function, and materials that are acceptable in peripheral tissue engineering may not be acceptable for central nervous system applications. Third, digital twin models depend heavily on data quality, completeness, and interpretability; wearable and smartphone data are often noisy, biased, and influenced by non-disease factors, while biomarker measurements may vary across laboratories and time points. Fourth, the integration of these tools into clinical workflows faces practical barriers, including cost, training, infrastructure, and patient acceptance. Addressing these limitations will require iterative cycles of preclinical testing, clinical validation, and regulatory planning, with materials, models, and clinical endpoints developed in parallel rather than sequentially.

If these priorities are pursued together and their limitations addressed, precision psychiatry may become more than a data-driven refinement of diagnosis. It may become a genuinely translational field in which patient-specific modeling guides the design, timing, and evaluation of therapeutic interventions. Such a shift would align psychiatric innovation with broader advances in bioengineering, regenerative medicine, digital health, and advanced materials. The next major advances, in our view, will not come from optimizing algorithms and materials separately, but from integrating them around clinically meaningful psychiatric states and biologically plausible intervention pathways.

## Data Availability

The original contributions presented in the study are included in the article/supplementary material, further inquiries can be directed to the corresponding author.
